# Association of TM6SF2 rs58542926 gene polymorphism with the risk of non-alcoholic fatty liver disease and colorectal adenoma in Chinese Han population

**DOI:** 10.1186/s12858-019-0106-3

**Published:** 2019-02-06

**Authors:** Yuan Li, Shousheng Liu, Yuqiang Gao, Huan Ma, Shuhui Zhan, Yan Yang, Yongning Xin, Shiying Xuan

**Affiliations:** 1 Medical College of Qingdao University, Qingdao, 266071 China; 2 Department of Gastroenterology, Qingdao Municipal Hospital, 1 Jiaozhou Road, Qingdao, 266011 Shandong Province China; 3 Department of Liver Disease, Qingdao Municipal Hospital, Qingdao, 266011 China; 4 Central Laboratories, Qingdao Municipal Hospital, Qingdao, 266071 China; 5 Digestive Disease Key Laboratory of Qingdao, Qingdao, 266071 China

**Keywords:** TM6SF2, rs58542926, NAFLD, Colorectal adenomas

## Abstract

**Background:**

Genetic factors affect the risk of non-alcoholic fatty liver disease (NAFLD) and colorectal adenoma (CRA) importantly. Transmembrane protein 6 superfamily member 2 (TM6SF2) rs58542926 is a significant genetic susceptibility site for NAFLD. The relationships of *TM6SF2* rs58542926 with the risk of NAFLD and CRA in Chinese Han population were unclear. The aim of this study was to investigate the association of *TM6SF2* rs58542926 with the risk of NAFLD and CRA, and the effect of CRA on *TM6SF2* rs58542926 carried NAFLD patients.

**Results:**

A total of 839 Chinese Han population were included in this retrospective study. *TM6SF2* rs58542926 polymorphism was genotyped in B-type ultrasonography proven NAFLD patients with or without CRA, CRA patients and healthy controls, using polymerase chain reaction. Serum lipid profiles were determined using biochemical methods. Statistical analyses were performed using SPSS statistical software, version 16.0 for mac. There was a significant difference in the distribution of genotype and allele of *TM6SF2* rs58542926 in NAFLD and NAFLD&CRA patients compared to controls. The CT + TT genotypes were tightly associated with the risk of NAFLD and NAFLD&CRA. *TM6SF2* rs58542926 T allele promotes the abnormal regulation of lipids metabolism and liver injury in NAFLD patients and NAFLD&CRA patients. CRA aggravates the clinical performance of NAFLD in T allele carriers.

**Conclusions:**

We demonstrated the significant association between *TM6SF2* rs58542926 polymorphism and the risk of NAFLD and NAFLD&CRA in a Chinese Han population. The *TM6SF2* rs58542926 T allele promotes the abnormal regulation of lipid profiles and liver injury in NAFLD patients, NAFLD&CRA patients, and overall subjects.

## Background

NAFLD is one of the most common chronic liver diseases in the world [1]. NAFLD is the manifestation of metabolic syndrome in liver, which comprises of simple non-alcoholic fatty liver (NAFL), non-alcoholic steatohepatitis (NASH), fibrosis, and cirrhosis, even the hepatocellular carcinoma (HCC) [2]. In Europe, the prevalence of NAFLD increases year by year due to the risk factors such as obesity and aging [3]. An eight-year follow-up study in China showed that NAFLD was prevalent in common population and the increased tendency of NAFLD was obviously [4].

In recent years, genome wide association studies (GWAS) have been conducted to explore the significant SNP (single nucleotide polymorphism) sites in genome which were associated NAFLD and lipid metabolism. Several significant genetic susceptibility genes such as *PNPLA3*, *KLF6*, *GCKR*, *LYPLAL1*, had been proven the key roles in the development of NAFLD in different regions and ethnicities [5–10]. *TM6SF2* rs58542926 is a substitution of guanine by adenine in nucleotide 499, that leads to the replacement of glutamic acid by lysine in amino acid residue 167 (E167K). In 2014, the significance of *TM6SF2* rs58542926 polymorphism in NAFLD was found for the first time [11]. Subsequent studies had proven that *TM6SF2* rs58542926 was a significant risk factor for the development of NAFLD. Chen et al. systematic reviewed the genetic susceptibility of *TM6SF2* rs58542926 in NAFLD [12]. Giovanni et al. proved that *TM6SF2* rs58542926 could affect nutrient oxidation, glucose homeostasis, and postprandial lipoprotein, adipokine in NAFLD patients [13]. In addition, the effect of *TM6SF2* rs58542926 on serum lipid levels in obese children was investigated by Grandone et al., the results showed that *TM6SF2* rs58542926 was significant associated with the lower level of low density lipoprotein cholesterol, and could promote liver injury in obese children [14]. In China, the key effect of *TM6SF2* rs58542926 on NAFLD in Shanghai district and Hong Kong was reported by Wong and Wang, that provided a primary evidence for the key role of *TM6SF2* rs58542926 in Asian especially in Chinese [15, 16].

Colorectal cancer (CRC) is one of the most prevalent malignancies in the world, the incidence of CRC has risen steadily in recent years [17, 18]. CRA is a benign precursor of CRC, polyps - adenoma - cancer sequence has been recognized as the typical patterns of CRC [19, 20]. Accumulated evidences have shown that NAFLD and metabolic syndrome were tightly associated with CRA, and abnormal lipid metabolism was the significant characteristic in the patients with NAFLD and CRA [21–24]. Some polymorphism sites such as *ADH1B* Arg47His, *NAT1* rs65867, and the gene polymorphisms in WNT6 and WNT10A in patients with CRA had been studied, the results suggested that the risk of CRA was markedly associated with gene genetic susceptibility [25–27]. In view of the tight association of CRA and NAFLD, the role of *TM6SF2* rs58542926 in the risk of CRA was unknown. The purpose of this study was to investigate the relationship of *TM6SF2* rs58542926 with the risk of NAFLD and CRA in Qingdao district of China and explore the effect of CRA on the *TM6SF2* rs58542926 carried NAFLD patients.

## Methods

### Subjects

This study was approved by the ethics committee of Qingdao municipal hospital and strictly in accordance with the Helsinki declaration and its appendices [28]. Each patient was signed the written informed consent before participating in this study. All the subjects were recruited in the Health examination center and department of gastroenterology of Qingdao municipal hospital from March 2016 to March 2018, and all the subjects are the unrelated Chinese Han population. The essential information such as name, gender, age, was obtained using a standard questionnaire.

All patients were subjected to the colonoscopy by three experienced endoscopists with at least 5 years’ experience in colonoscopy examination at the Qingdao municipal hospital. Before colonoscopy examination, subjects took the polyethylene glycol electrolyte powder orally to prepare the bowel. The quality of bowel preparation was evaluated according to the intestinal residual contents and surface seen as good (more than 95% surface seen and without or small volume of clear liquid), poor (less than 90% surface seen, and semi-solid stool presented that could not be suctioned or washed away), and fair (between good and poor). Endoscope (OLYMPUS 260, Japan) reached caecum was defined as a complete colonoscopy examination. Subjects with an incomplete colonoscopy examination would be excluded from this study. The CRA were defined as a benign tumor originating from the glandular epithelium of colorectal mucosa, including colon adenoma and rectal adenoma. Subjects as below were excluded: 1) suffering from tumors or surgery in nearly 2 years; 2) suffering from inflammatory bowel disease or chronic liver disease or kidney disease; 3) possessing a history of heart failure; 4) with autoimmune disorders (rheumatoid arthritis, AIDS); 5) long-term use of immunosuppression preparations; 6) suffering from asthma, COPD or other pulmonary diseases; 7) familial polyposis.

The diagnosis of NAFLD was conducted according to the criteria of American association for the study of liver diseases (AASLD) [29]. All the NAFLD patients were diagnosed 10 times repeatedly at the same point by two experienced physicians. FibroScan CA*P* value was obtained automatically by taking the median. Patients with other causes such as high alcohol intake (males > 210 g/w, females > 140 g/w), autoimmune hepatitis, viral hepatitis, drug-induced hepatitis, various liver cirrhosis and alcoholic liver disease were also excluded. Healthy controls were confirmed by the same examinations at Qingdao municipal hospital. According to the diagnostic results, subjects were divided into four groups: NAFLD group, NAFLF & CRA group, CRA group, and healthy control group.

### Biochemical analyses

Blood sample was taken from the median vein of each subject with a 12-h overnight fasting and placed into the ethylene diamine tetraacetic acid (EDTA)-containing tube. The body mass index (BMI) of each subject was calculated equals to mass (kg)/height (m)^2^. For biochemical analyses, serum levels of fasting blood glucose (FBG), total cholesterol (TC), triglyceride (TG), low density lipoprotein cholesterol (LDL-C), high density lipoprotein cholesterol (HDL-C), aspartate aminotransferase (AST), alanine transaminase (ALT), uric acid (UA), were measured by standard clinical laboratory techniques (IChem-540 automatic biochemical analyzer, Shenzhen, China), respectively.

### Genomic DNA extraction and genotyping

Genomic DNA was extracted from blood sample using the Genomic DNA purification Kit (Beijing Bioteke Biotechnology, Beijing, China) as described by [30]. Polymerase chain reaction (PCR) was performed to test the genotype of *TM6SF2* rs58542926 of all the subjects using the primers: 5’-ACGTTGGATGTGAAGACCTTCATGCCAGCC-3′ and 5’-ACGTTGGATGGCACCATGGAAGGCAAATAC-3′. PCR amplification (Labnet, United States) was performed as the following program: 95 °C for 10 min, followed by 35 cycles: denaturation at 94 °C for 3 min, annealing at 58 °C for 1 min and extension at 72 °C for 1 min. All PCR products were resolved using 2% agarose gel electrophoresis at 120 V for 30 min and stained with ethidium bromide. DNA sequencing was performed to identify the genotype of *TM6SF2* rs58542926 using the ABI Prism sequence detection system ABI3730 (Foster city, CA, USA), and the SNP genotyping success rates were > 95%. All Genotyping were performed in blinded fashion.

### Data analysis

Statistical analyses were performed using SPSS (version 16.0 for Mac) statistical software as previously described [30]. The Hardy-Weinberg equilibrium between expected and observed genotype distribution and the distributions of genotype between patients and controls were analyzed by Pearson’s χ^2^ test.

Genotype and allele frequencies were investigated by counting the DNA sequencing data of each subject. Clinical and biochemical characteristics of each patient were shown as mean ± standard deviation (SD) and the differences of characteristics in different groups were tested using the χ^2^ test, student’s *t*-test or paired samples *t*-test. The association between polymorphism and presence/absence of NAFLD/CRA was evaluated by logistic regression analysis and estimated by the odds ratio (OR) with 95% confidence interval (CI). *P* < 0.05 was considered as statistically significant.

## Results

### Characteristics of the study population

A total of 839 subjects were included in this study, which including 201 patients (128 males and 73 females) with NAFLD, 188 patients (127 males and 61 females) with NAFLD&CRA, 211 patients (134 males and 77 females) with CRA, and 239 healthy control subjects (133 males and 106 females). The clinical characteristics of all the subjects were shown in the Table 1. As expected, the traditional risk factors of NAFLD, such as BMI, CAP, FPG, TG, AST, ALT, and UA were significant higher in the NAFLD group and NAFLD&CRA group compared to healthy control group (all *P* < 0.05). In CRA group, the serum levels of BMI, CAP, FPG, TG, and UA were significantly higher than in healthy control group (all *P* < 0.05). Compared to the CRA group, the higher serum levels of CAP and ALT in NAFLD group, and the higher serum levels of CAP and AST in NAFLD&CRA group were observed (all *P* < 0.05). No significant differences of all the clinical characteristics were observed between NAFLD group and NAFLD&CRA group (Table 1).

**Table 1:** Clinical and biochemical characteristics of each group patients^a^

Characteristic	NAFLD (*n* = 201)	NAFLD&CRA (*n* = 188)	CRA (*n* = 211)	Control (*n* = 239)
Age (yr)	54.80 ± 0.56	54.60 ± 0.48	55.10 ± 0.52	54.00 ± 0.78
BMI (kg/m^2^)	24.56 ± 1.28*	25.45 ± 2.07*	24.12 ± 1.43*	23.05 ± 0.67
CAP (dB/m)	242.56 ± 31.42*^@^	245.64 ± 29.63*^@^	230.66 ± 32.33*	201.23 ± 28.67
FPG (mmol/L)	5.01 ± 0.45*	5.24 ± 0.89*	5.09 ± 0.78*	4.78 ± 0.99
TC (mmol/L)	5.49 ± 0.95	5.56 ± 0.87	5.46 ± 0.78	5.43 ± 0.79
TG (mmol/L)	1.96 ± 1.23*	1.98 ± 1.34*	1.89 ± 1.27*	1.48 ± 1.06
LDL-C (mmol/L)	3.32 ± 0.45	3.31 ± 0.79	3.28 ± 0.67	3.20 ± 0.77
HDL-C (mmol/L)	1.33 ± 0.32	1.32 ± 0.37	1.37 ± 0.56	1.38 ± 0.41
AST (U/L)	39.60 ± 8.30*	40.50 ± 9.2*^@^	34.50 ± 8.50	31.40 ± 7.30
ALT (U/L)	37.20 ± 9.90*^@^	39.90 ± 10.30*	31.80 ± 11.90	30.90 ± 10.10
UA (μmol/L)	368.76 ± 80.32*	370.58 ± 67.92*	369.61 ± 84.26*	340.56 ± 75.67

*Abbreviations*: *NAFLD* non-alcoholic fatty liver disease, *CRA* colorectal adenoma, *BMI* body mass index, *CAP* controlled attenuation parameter, *FPG* fasting plasma glucose, *TC* total cholesterol, *TG* triglyceride, *LDL-C* low-density lipoprotein cholesterol, *HDL-C* high-density lipoprotein cholesterol, *AST* aspartate aminotransferase, *ALT* alanine aminotransferase, *UA* uric acid

^*^compared with the control group, *P* < 0.05; ^@^ compared with CRA group, *P* < 0.05

^a^Data are presented as mean ± SD

### Distribution of genotype and allele

The genotype distributions of *TM6SF2* rs58542926 were in consistence with the Hardy-Weinberg (H-W) equilibrium in all the four groups (all *P* > 0.05) (Table 2). The variant rs58542926T allele frequencies in NAFLD group, NAFLD&CRA group, CRA group, and healthy control group were 3.2, 3.5, 1.7, and 1.0%, respectively (Table 3). To ensure the accuracy of our genotyping, 100 samples were randomly selected for reverse sequencing, the success rate of duplicated genotyping was 100%. There were significant differences of rs58542926 genotype distributions in the NAFLD group vs control group, NAFLD&CRA group vs control group, and NAFLD group vs CRA group (all *P* < 0.05), no significant differences of rs58542926 genotype distributions were observed in the CRA group vs control group, NAFLD&CRA group vs NAFLD group, and NAFLD&CRA vs CRA group. Similar, the allele distributions of rs58542926 in NAFLD group vs control group, NAFLD&CRA group vs control group were significant different (both *P* < 0.05), no significant differences of rs58542926 allele distributions were observed in the CRA group vs control group, NAFLD&CRA group vs NAFLD group, NAFLD&CRA vs CRA group, and NAFLD group vs CRA group (Table 3). Presence of CT + TT genotype markedly associated the risk of NAFLD (OR: 1.464, 95%CI: 1.300–1.599, *P* = 0.013), the significant association of CT + TT genotype with the risk of NAFLD&CRA was also observed in NAFLD&CRA group (OR: 1.235, 95%CI: 1.083–1.801, *P* = 0.027). Presence of CT + TT genotype did not associate with the risk of CRA (OR: 1.053, 95% CI: 0.986–1.599, *P* = 0.231). After adjusting for gender, age, and BMI, the association of *TM6SF2* rs58542926 CT + TT genotype with the risk of NAFLD and NAFLD&CRA were still significant (OR: 1.368, 95% CI: 1.113–1.504, *P* = 0.025; OR: 1.129, 95% CI: 1.002–1.768, *P* = 0.038, respectively) (Table 4).

**Table 2:** Results of the Hardy-Weinberg (H-W) Equilibrium^a^

Group	χ^**2**^	*P* value
NAFLD	0.2245	0.64
NAFLD&CRA	0.2411	0.62
CRA	0.0600	0.81
Control	0.0267	0.87

^a^Data were compared by chi-square test

**Table 3:** Distribution of genotypes and allele frequencies of *TM6SF2* rs58542926 in each group^a,b^

Group	Genotype					Allele			
	CC	CT	TT	χ^2^	*P* value	C	T	χ^2^	*P* value
NAFLD	188 (93.5%)	13 (6.5%)	0 (0.0%)	5.328	0.021	389 (96.8%)	13 (3.2%)	5.238	0.022
Control	234 (97.9%)	5 (2.1%)	0 (0.0%)			474 (98.9%)	5 (1.1%)		
NAFLD&CRA	175 (93.1%)	13 (6.9%)	0 (0.0%)	6.062	0.014	363 (96.5%)	13 (3.5%)	5.954	0.015
Control	234 (97.9%)	5 (2.1%)	0 (0.0%)			474 (98.9%)	5 (1.1%)		
CRA	204 (96.7%)	7 (3.3%)	0 (0.0%)	0.648	0.421	415 (98.3%)	7 (1.7%)	0.646	0.422
Control	234 (97.9%)	5 (2.1%)	0 (0.0%)			474 (98.9%)	5 (1.1%)		
NAFLD&CRA	175 (93.1%)	13 (6.9%)	0 (0.0%)	0.031	0.860	363 (96.5%)	13 (3.5%)	0.030	0.862
NAFLD	188 (93.5%)	13 (6.5%)	0 (0.0%)			389 (96.8%)	13 (3.2%)		
NAFLD&CRA	175 (93.1%)	13 (6.9%)	0 (0.0%)	2.702	0.100	363 (96.5%)	13 (3.5%)	2.633	0.105
CRA	204 (96.7%)	7 (3.3%)	0 (0.0%)			415 (98.3%)	7 (1.7%)		
NAFLD	188 (93.5%)	13 (6.5%)	0 (0.0%)	4.054	0.044	389 (96.8%)	13 (3.2%)	2.439	0.118
CRA	204 (96.7%)	7 (3.3%)	0 (0.0%)			415 (98.3%)	7 (1.7%)		

^a^Data were compared by chi-square test

^b^Values are expressed as No. (%)

**Table 4:** Association of genotypes with NAFLD, NAFLD&CRA and CRA groups^a^

Group	Unadjusted			Adjusted		
	Genotype	OR (95% *CI*)	*P* value	Genotype	OR (95% *CI*)	*P* value
NAFLD	CC	1	0.013	CC	1	0.025
	CT + TT	1.464 (1.300–1.599)		CT + TT	1.368 (1.113–1.504)	
NAFLD&CRA	CC	1	0.027	CC	1	0.038
	CT + TT	1.235 (1.083–1.801)		CT + TT	1.129 (1.002–1.768)	
CRA	CC	1	0.231	CC	1	0.202
	CT + TT	1.053 (0.986–1.599)		CT + TT	1.209 (0.954–1.783)	

^a^The multiple-logistic regression model was adjusted for gender, age, and BMI

### Association of TM6SF2 rs58542926 T allele with the clinical parameters in each group and overall subjects

To investigate whether the *TM6SF2* rs58542926 T allele are correlated with clinical parameters, we compared the clinical parameters in T allele carriers and non-carriers in each group and overall subjects. As the results shown in the Table 5, in NAFLD group, T allele carriers had the higher levels of CAP and ALT, and lower level of TG than non-carriers (all *P* < 0.05). In NAFLD&CRA group, the higher levels of CAP, AST, and ALT, and lower levels of TC, TG, and LDL-C were observed in T allele carriers when compared to non-carriers (all *P* < 0.05). However, no significant differences of all the clinical parameters were observed between the T allele carriers and non-carriers in the CRA group and healthy control group (all *P* > 0.05). In overall subjects, T allele carriers possessed the higher CAP value and serum AST level, and the lower serum TG level than non-carriers (Table 6).

**Table 5:** Clinical characteristics of *TM6SF2* rs58542926 T carriers and non-carriers in each group^a^

	NAFLD			NAFLD&CRA		
	Carriers (*n* = 13)	Non-carriers (*n* = 188)	*P* value	Carriers (*n* = 13)	Non-carriers (*n* = 175)	*P* value
Age (yr)	53.80 ± 0.46	55.00 ± 0.51	0.534	54.90 ± 0.61	54.30 ± 0.49	0.411
BMI (kg/m^2^)	24.99 ± 1.36	23.95 ± 1.23	0.957	24.98 ± 1.98	26.12 ± 2.02	0.053
CAP (dB/m)	262.34 ± 32.65	239.11 ± 37.76	0.031	260.21 ± 22.52	240.46 ± 21.34	0.001
FPG (mmol/L)	4.89 ± 0.98	5.13 ± 0.12	0.163	5.21 ± 0.32	5.26 ± 0.79	0.341
TC (mmol/L)	5.01 ± 0.67	5.63 ± 0.83	0.352	4.80 ± 0.98	5.61 ± 0.78	0.013
TG (mmol/L)	1.49 ± 1.21	2.09 ± 0.95	0.031	1.50 ± 1.39	2.12 ± 1.09	0.001
LDL-C (mmol/L)	3.38 ± 0.37	3.29 ± 0.41	0.571	3.12 ± 0.62	3.36 ± 0.70	0.039
HDL-C (mmol/L)	1.39 ± 0.43	1.24 ± 0.12	0.368	1.30 ± 0.32	1.35 ± 0.37	0.276
AST (U/L)	44.40 ± 7.90	37.30 ± 7.60	0.067	46.30 ± 8.90	38.70 ± 8.70	0.048
ALT (U/L)	43.70 ± 8.90	35.76 ± 9.10	0.039	45.80 ± 10.40	37.80 ± 9.40	0.043
UA (μmol/L)	363.34 ± 81.36	373.43 ± 78.02	0.632	372.51 ± 70.91	367.48 ± 61.32	0.652
	CRA			Control		
	Carriers (*n* = 7)	Non-carriers (*n* = 204)	*P* value	Carriers (*n* = 5)	Non-carriers (*n* = 234)	*P* value
Age (yr)	55.90 ± 0.54	54.90 ± 0.48	0.853	53.80 ± 0.57	54.20 ± 0.65	0.967
BMI (kg/m^2^)	24.38 ± 1.38	23.85 ± 1.56	0.574	22.98 ± 0.91	23.13 ± 0.77	0.968
CAP (dB/m)	240.66 ± 34.73	227.66 ± 30.67	0.195	210.45 ± 30.67	199.48 ± 25.76	0.698
FPG (mmol/L)	5.04 ± 0.45	4.79 ± 0.71	0.837	4.99 ± 1.03	4.69 ± 0.68	0.753
TC (mmol/L)	5.29 ± 0.98	5.54 ± 0.63	0.111	5.27 ± 0.76	5.74 ± 0.64	0.386
TG (mmol/L)	1.76 ± 1.28	1.92 ± 1.37	0.093	1.36 ± 0.98	1.57 ± 1.21	0.215
LDL-C (mmol/L)	3.23 ± 0.75	3.30 ± 0.63	0.265	2.92 ± 0.97	3.31 ± 0.67	0.470
HDL-C (mmol/L)	1.47 ± 0.63	1.33 ± 0.59	0.097	1.57 ± 0.31	1.31 ± 0.54	0.865
AST (U/L)	36.90 ± 10.80	33.50 ± 7.50	0.683	30.60 ± 6.30	32.70 ± 7.70	0.461
ALT (U/L)	30.60 ± 9.90	32.80 ± 13.10	0.473	26.90 ± 8.10	32.90 ± 10.30	0.643
UA (μmol/L)	358.61 ± 81.53	379.61 ± 84.98	0.352	335.36 ± 67.69	351.55 ± 73.64	0.198

*Abbreviations*: *NAFLD* non-alcoholic fatty liver disease, *CRA* colorectal adenoma, *BMI* body mass index, *CAP* controlled attenuation parameter, *FPG* fasting plasma glucose, *TC* total cholesterol, *TG* triglyceride, *LDL-C* low-density lipoprotein cholesterol, *HDL-C* high-density lipoprotein cholesterol, *AST* aspartate aminotransferase, *ALT* alanine aminotransferase, *UA* uric acid

^a^Values are expressed as mean ± SD and compared by Student’s *t*-test

**Table 6:** Clinical characteristics of *TM6SF2* rs58542926 T carriers and non-carriers in overall subjects^a^

Characteristic	CC (*n* = 794)	CT + TT (*n* = 45)	*P* Value
Age (yr)	54.60 ± 0.49	54.70 ± 0.48	0.863
BMI (kg/m^2^)	24.26 ± 1.40	24.33 ± 1.41	0.841
CAP (dB/m)	226.68 ± 28.88	243.42 ± 30.14	0.034
FPG (mmol/L)	4.97 ± 0.58	5.03 ± 0.81	0.741
TC (mmol/L)	5.63 ± 0.72	5.09 ± 0.85	0.175
TG (mmol/L)	1.93 ± 1.15	1.53 ± 1.22	0.029
LDL-C (mmol/L)	3.32 ± 0.60	3.16 ± 0.68	0.492
HDL-C (mmol/L)	1.31 ± 0.41	1.43 ± 0.42	0.429
AST (U/L)	39.66 ± 8.50	43.93 ± 7.90	0.042
ALT (U/L)	34.82 ± 10.50	36.57 ± 9.30	0.093
UA (μmol/L)	368.03 ± 74.49	357.46 ± 75.37	0.735

*Abbreviations*: *NAFLD* non-alcoholic fatty liver disease, *CRA* colorectal adenoma, *BMI* body mass index, *CAP* controlled attenuation parameter, *FPG* fasting plasma glucose, *TC* total cholesterol, *TG* triglyceride, *LDL-C* low-density lipoprotein cholesterol, *HDL-C* high-density lipoprotein cholesterol, *AST* aspartate aminotransferase, *ALT* alanine aminotransferase, *UA* uric acid

^a^Values are expressed as mean ± SD and compared by Student’s *t*-test

## Discussion

NAFLD as the most prevalent chronic liver disease is affected by many risk factors such as obesity, insulin resistance, oxidative stress, and genetic factors [31, 32]. Increased understanding of the underlying mechanism in genetics for the development of NAFLD was implemented [33, 34]. *TM6SF2* is a novel genetic susceptibility gene and accumulated attentions have been paid to the association of *TM6SF2* rs58542926 with the risk of NAFLD [12, 35], but the studies conducted in Chinese Han population were remain insufficient. Some reports showed that CRA was tightly associated with the risk of NAFLD, and the incidence of NAFLD combined with CRA was increasing in recent years [36, 37]. In this study, we investigated the association of *TM6SF2* rs58542926 polymorphism with the risk of NAFLD in Han population of Qingdao district in China, and explored the effect of CRA on the *TM6SF2* rs58542926 carried NAFLD patients. The results in this study showed that patients with NAFLD and NAFLD&CRA had the higher BMI and CAP values and serum levels of FGP, TG, AST, ALT, and UA than healthy controls. In additional, the higher BMI and CAP values and serum levels of FPG, TG, and UA were observed in patients with CRA compared to healthy controls. In Chinese Han population, *TM6SF2* rs58542926 was significant associated with the risk of NAFLD and NAFLD&CRA, but no with the risk of CRA.

NAFLD is the hepatic manifestation of metabolic syndrome, the abnormal regulation of glucose and lipid metabolism are existed in the NAFLD patients [38]. In recent years, with the development of diagnostic methods for NAFLD, transient elastography (FibroScan®, TE) has been used as an accurate non-invasive diagnostic method, the degrees of hepatic steatosis could be reflected by the CAP values [39, 40]. In our study, the typical performance of metabolic syndrome such as the higher BMI and CAP values, higher serum levels of FPG, TG, AST, ALT, and UA were observed in the NAFLD patients and NAFLD&CRA patients. Accumulated evidences have shown that the risk of NAFLD was tightly associated with CRA. Sang et al. found that NAFLD in CRA group was significantly higher than in healthy control group, and the increased risk of NAFLD was more serious in patients with a higher prevalence of colorectal adenomatous polyps [22]. Interestingly, we found that the BMI and CAP values, and the serum levels of FPG, TG, and UA were markedly higher in the CRA patients than in healthy controls, which were consist with the previous studies. Compared to the CRA patients, the NAFLD patients and NAFLD&CRA patients had the higher CAP values, ALT, and AST levels, suggested that CRA acts as a risk factor for NAFLD, but could not directly lead to the development of NAFLD and NAFLD-related liver injury. No significant differences of clinical characteristics between NAFLD patient and NAFLD&CRA patients were observed, showed that CRA did not contribute to the progression of NAFLD patients.

Since the *TM6SF2* rs58542926 was found, the genetic susceptibility of this variant in NAFLD had been studied by many researchers. Increasing evidences have proven that *TM6SF2* rs58542926 was an independent risk factor for NAFLD [15, 41, 42]. In our study, the *TM6SF2* rs58542926 genotype distributions were significant different in NAFLD group, NAFLD&CRA group compared to healthy control group. Besides, the genotype distributions were also different in NAFLD group and CRA group. The allele distributions of *TM6SF2* rs58542926 C and T in NAFLD group and NAFLD&CRA group were significant different compared to healthy control group. Multiple-logistic regression analysis showed that CT + TT genotype was significant associated with the risk of NAFLD and NAFLD&CRA, but no associated with CRA. These results suggested that *TM6SF2* rs58542926 was a significant risk factor of NAFLD. CRA did not markedly affect the distributions of genotype and allele in NAFLD patients.

Hepatic TG deposition leads to the decreased serum TG level, which is the typical symptom of NAFLD patients [43]. Several studies had investigated the effects of *TM6SF2* rs58542926 T allele on the lipid profiles. Eeva et al. conducted a study in 24,925 European populations and found that the plasma concentrations of LDL, fatty acids, TG and TC in various forms of lipoproteins in *TM6SF2* rs58542926 T allele carriers were significant lower than in control group [44]. Elizabeth et al. confirmed that in many aspects such as crowd, animal and cell, T allele could reduce the fasting plasma lipids and postprandial triglycerides [45]. In our study, we investigated the effect of *TM6SF2* rs58542926 T allele on the clinical characteristics in each group. In NAFLD patients, the higher CAP value and serum ALT level, and the lower serum TG level were observed in T allele carriers. The higher CAP value and serum AST and ALT levels, and the lower serum levels of TC, TG, and LDL-C were observed in the T allele carriers of NAFLD&CRA. As the AST and ALT are the significant liver injury biomarkers [46], our results suggested that *TM6SF2* rs58542926 T allele was associated with the hepatic TG accumulation, therefore leads to the decreased serum lipid profiles, and promotes the liver injury in NAFLD patients. In NAFLD&CRA patients, *TM6SF2* rs58542926 T allele also tightly associated with the abnormal regulation of lipids metabolism and liver injury, and the presence of CRA aggravates the clinical performance of NAFLD.

## Conclusions

In summary, we investigated the relationship of *TM6SF2* rs58542926 polymorphism with the risk of NAFLD and CRA, and the effect of CRA on the *TM6SF2* rs58542926 carried NAFLD patients in Han population of Qingdao district in China. Our results showed that *TM6SF2* rs58542926 polymorphism was tightly associated with the risk of NAFLD and NAFLD&CRA, but no associated with the risk of CRA. *TM6SF2* rs58542926 T allele promotes the liver injury and abnormal regulation of lipids metabolism in NAFLD patients, NAFLD&CRA patients, and overall subjects.

## Abbreviations

AASLD: American association for the study of liver disease
ALT: Alanine transaminase
AST: Aspartate aminotransferase
BMI: Body mass index
CI: Confidence interval
CRA: Colorectal adenoma
CRC: Colorectal cancer
FBG: Fasting blood glucose
GWAS: Genome wide association studies
HCC: Hepatocellular carcinoma
HDL: High density lipoprotein cholesterol
LDL: Low density lipoprotein cholesterol
NAFL: Non-alcoholic fatty liver
NAFLD: Non-alcoholic fatty liver disease
NASH: Non-alcoholic steatohepatitis
OR: Odds ratio
PCR: Polymerase chain reaction
SNP: Single nucleotide polymorphism
TC: Total cholesterol
TG: Triglyceride
TM6SF2: Transmembrane protein 6 superfamily member 2
UA: Uric acid
